# The simplified Chinese version of the Knee Injury and Osteoarthritis Outcomes Score (KOOS) in individuals with knee osteoarthritis for mainland China: the study of reliability and validity

**DOI:** 10.1186/s41687-023-00619-2

**Published:** 2023-07-27

**Authors:** Liying Yang, Jatuporn Suttiwong, Yanfen Fu, Komsak Sinsurin

**Affiliations:** 1 Biomechanics and Sports Research unit, Faculty of Physical Therapy, Mahidol University, 999 Phuttamonthon 4 Road, Salaya, Nakhon Pathom, 73170 Thailand; 2 Functional and Disability Questionnaire Research unit, Faculty of Physical Therapy, Mahidol University, 999 Phuttamonthon 4 Road, Salaya, Nakhon Pathom, 73170 Thailand; 3 College of Nursing, Dali University, Dali, China

**Keywords:** Reliability, Validity, Simplified chinese version of the knee Injury and Osteoarthritis Outcome score, KOOS, Health-related quality of life

## Abstract

**Background:**

The Knee Injury and Osteoarthritis Outcomes Score (KOOS) is a free clinical tool commonly used to evaluate the symptoms and functional status of patients with knee injury. For people who speak Chinese, the Hong Kong Chinese and Singapore Chinese versions are preferred. However, variations in the Chinese language and culture are influenced by the country’s geography. KOOS for Mainland China has not been reported. Therefore, the current study was to cross-culturally translate the original English version into a simplified Chinese version and to investigate its psychometric properties.

**Methods:**

The simplified Chinese KOOS was obtained through forward-backward translation according to appropriate guidelines. A total of 158 individuals with knee osteoarthritis (KOA) were recruited from 13 hospitals in China to examine the psychometric properties. The test-retest questionnaire was performed at an interval of 5–7 days. Test-retest reliability and internal consistency were evaluated using the intraclass correlation coefficient (ICC) and Cronbach’s alpha, respectively. The data of the first test were used to analyse the construct validity of the simplified Chinese KOOS and Chinese SF-36 through convergent and discriminant validity using Spearman’s correlation coefficient.

**Results:**

Cross-cultural translation exhibited minor cultural differences, and the questionnaire was well understood by the patients. The data from 128 patients, used for the test-retest reliability study, showed good to excellent reliability, with an ICC of 0.808–0.976 for all KOOS subscales. The Cronbach’s alpha for all subscales ranged from 0.757 to 0.970, indicating acceptable internal consistency. There was a low-to-high correlation between the five domains of the simplified Chinese version of the KOOS and all domains of the SF-36 in construct validity.

**Conclusion:**

The simplified Chinese KOOS demonstrated acceptable reliability and validity. In clinical practice and research, this version can help provide valuable information on health-related quality of life for Chinese individuals with KOA in mainland China.

## Background

Knee osteoarthritis (KOA), as a result of cartilage damage, is a major health problem worldwide caused by pain, swelling, stiffness, and disability [1]. In addition to structural and functional limitations, pain and disability affect social connections, interpersonal relationships, and emotional wellbeing. Subsequent impairment in quality of life (QOL) has also been reported [2, 3].

A self-administered questionnaires reported by patients is commonly used as clinical tools to describe functional status and QOL, to evaluate clinical outcomes, and to prevent biased evaluations by observers [4]. The Knee Injury and Osteoarthritis Outcomes Score (KOOS) is a free clinical tool commonly used to evaluate the symptoms and functional status of patients with knee injury. The complete version is available at www.koos.nu. It was developed in American English [5] and Swedish [6] by Professor Ewa Maria Roos in 1998, and is based on the Western Ontario and McMaster Universities Osteoarthritis Index (WOMAC) [5, 7].

The KOOS has been found to be a reliable and effective outcome indicator in different groups of patients with knee joint injuries and following surgery, such as chondral lesions [8], anterior cruciate ligament reconstruction [9], cartilage injury [10, 11], KOA [12], and total knee replacement [13]. Garratt et al. [14] reviewed knee-related patient outcome measurements and suggested that the KOOS is the most appropriate assessment tool for knee-related health problems. The reliability, validity, and responsiveness of the KOOS are superior to those of other tools, whether used in clinical or research settings [5, 6, 15].

Currently, the KOOS has been published in more than 50 languages and is used in various cultures. For people who speak Chinese, the Hong Kong Chinese [16] and Singapore Chinese [17] versions are preferred, especially when applied to clinical practice and research in Hong Kong and Singapore, respectively. However, variations in the Chinese language and culture are influenced by the country’s geography. Cantonese is written and spoken by the Hong Kong and Guangdong Provinces. However, Mandarin is the official language used by most Chinese people on the mainland China. In terms of writing, “knee joint swelling” is written as “膝關節腫脹” in Cantonese, but as “膝关节肿胀” in simplified Mandarin Chinese. The pronunciations of “knee joint swelling” in Cantonese and simplified Chinese are completely different. The previous KOOS version related to Chinese cannot be perfectly used by mainland Chinese people due to different writing, spelling rules and cross-cultural place difference. Therefore, we aimed to cross-culturally translate the original English version to a simplified Chinese version and to investigate the psychometric properties of individuals with KOA in mainland China. We hypothesized that a simplified Chinese KOOS has an acceptable range of reliability and validity in clinical practice. High test-retest reliability was expected between 1st and 2nd assessments in people with knee OA. When comparing the Chinese KOOS and SF-36 scales to measure the same or similar construct (convergent construct validity), we expected high correlations between the Chinese KOOS pain subscale and the SF-36 body pain, and Chinese KOOS QOL/ADL subscale and SF-36 physical functioning subscale. Low correlations between the Chinese KOOS all subscales and the SF-36 mental health subscale were expected to measure the different construct (discriminant construct validity).

## Methods

The authors obtained permission from Professor Ewa Maria Roos, an original developer, to cross-culturally translate the simplified Chinese KOOS before conducting this research. The research protocol was approved by the Mahidol University Central Institutional Review Board (MU-CIRB 2022/157.3005).

### Translation and cross-cultural adaptation

**Fig. 1:**
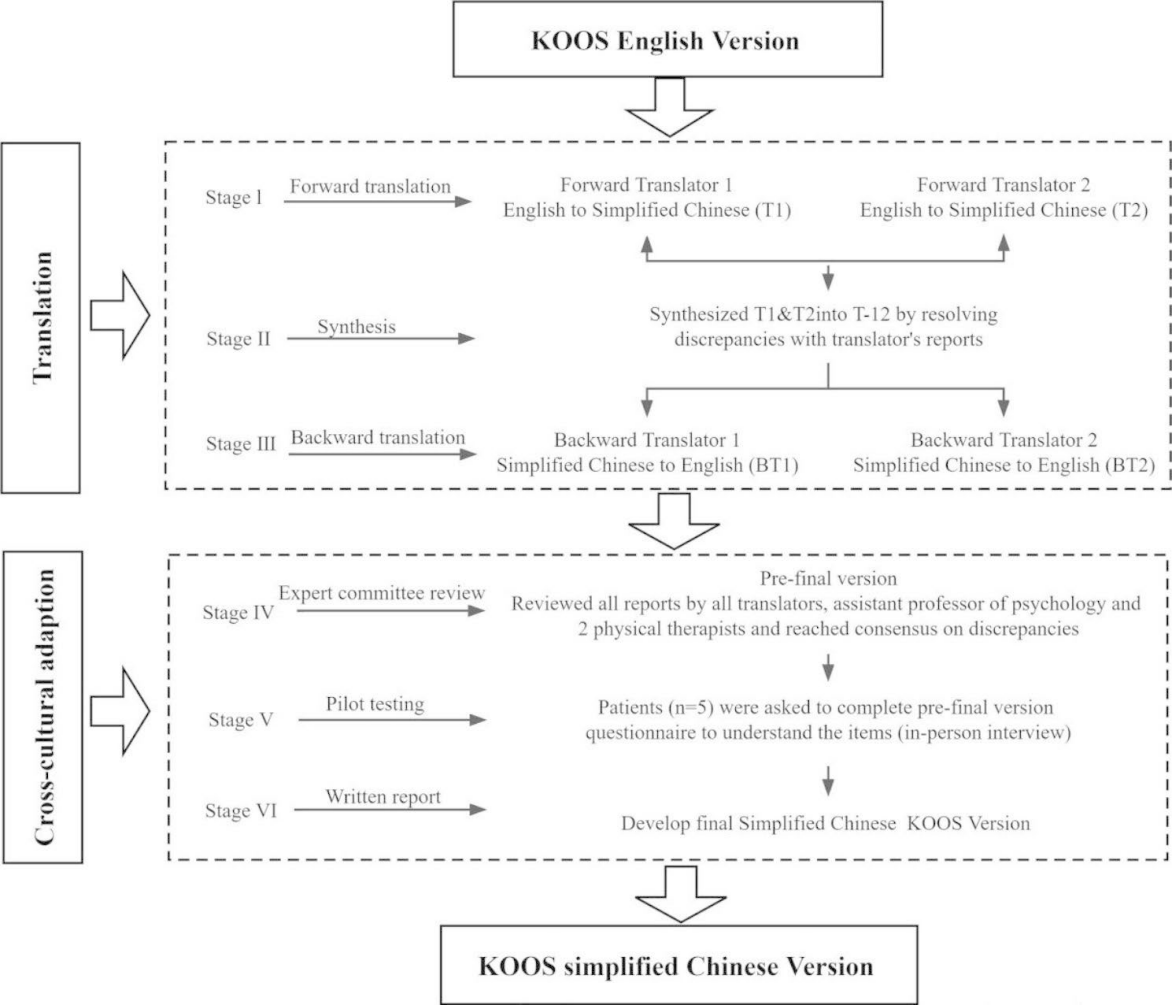
Flowchart diagram of translation and cross-cultural adaptation procedure. KOOS, Knee Injury and Osteoarthritis Outcomes Score

The simplified Chinese KOOS was translated according to the recommendations of Beaton’s health-related translation guide [21] (Fig. 1). Two native Chinese translators (a professional English teacher and a health professional) translated the original English KOOS into a simplified Chinese KOOS. The obtained version of the Chinese translation was translated back into American English by two Americans (who have lived in China for more than 10 years) without knowing the original text. To reach a consensus, all translators, two physiotherapists, a psychologist, and a Chinese language writer adjusted the cultural vocabulary and spoken language and discussed the forward and backward translation to form the simplified Chinese KOOS as a pre-final version. Five patients with KOA were recruited for the pilot study of this version to check their understanding. After the pilot study, a final version of the simplified Chinese KOOS was obtained.

### Psychometric property testing

**Fig. 2:**
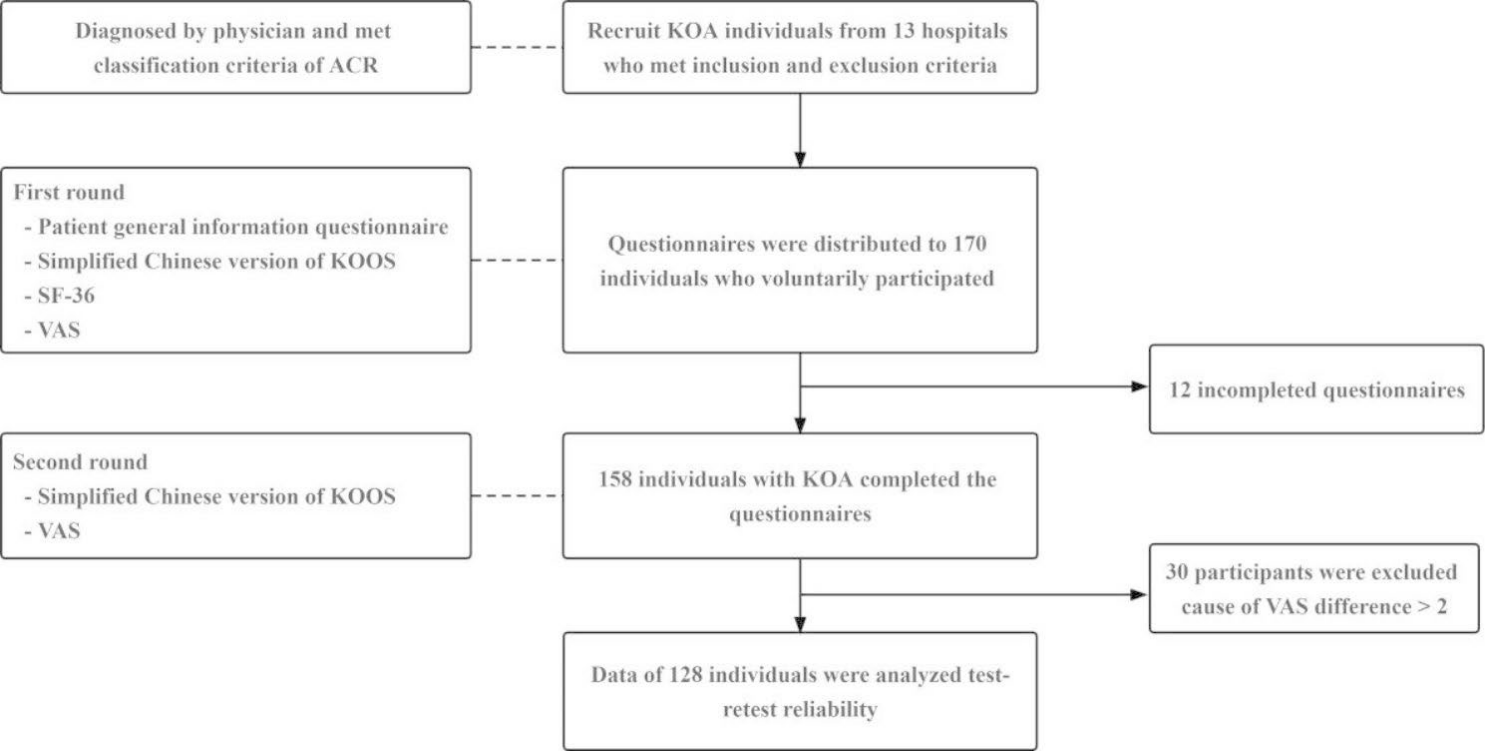
Flowchart of participant enrolment. ACR, American College of Rheumatology classification criteria; VAS, Visual Analogue Scale; SF-36, Short Form-36 Health Survey

The test-retest reliability, internal consistency, and construct validity (convergent and discriminant validity) of the simplified Chinese KOOS were examined. Data were collected from August to September 2022 from 158 individuals with KOA who voluntarily participated in this study (Fig. 2). We recruited individuals with KOA from 13 hospitals: Lijiang People’s Hospital; Ninglang County People’s Hospital; Ninglang County Hospital of Traditional Chinese Medicine; Yongsheng County People’s Hospital; Dali Prefecture People’s Hospital; The First Affiliated Hospital of Dali University; Dali First People’s Hospital; The Second People’s Hospital of Dali City; Xiangyun County Hospital; Heqing County Hospital; Xishuangbanna People’s Hospital; Qujing First People’s Hospital; and Lushui County People’s Hospital.

In each of the 13 hospitals, a volunteer physiotherapist with at least three years of experience in the orthopaedic field was selected for training. The training content included familiarisation with all scales in the study: KOOS; SF-36; visual analogue scale (VAS); and general information questionnaire. When a patient encountered problems during the form completion process, the physiotherapist could actively respond to the patient’s questions. Additionally, the physiotherapist was able to master the American College of Rheumatology (ACR) classification criteria to re-evaluate the patient as a KOA patient, maintain patient confidentiality, and check the integrity of the questionnaire on-site and code it.

Prior to completing the questionnaires, a trained physical therapist communicated with the patient. This was to ensure that the patient was aware of the content of the questionnaire, the requirements, and the time required to complete it, and to ensure that all information would be kept confidential.

The participants were native Chinese speakers who could read, understand, and complete the Chinese questionnaire. They were diagnosed with KOA by a physician and met at least three of the ACR classification criteria [18–20]. Participants were excluded if they had psychiatric disorders, neurological disorders, fractures or major surgery of the lower limbs, knee intra-articular injections in the previous six months, inflammatory arthritis, or knee inflammatory signs.

During the first testing visit, all participants were asked to complete the simplified Chinese KOOS, Chinese SF-36 questionnaire, VAS of pain, and general information questionnaire. The general information questionnaire consisted of asking the patient about the duration of the disease, affected leg, education level, and age. To re-administer the simplified KOOS, a period of 5–7 days between the first and second administrations was preferred. This time interval was considered sufficient to prevent answer recall and was sufficiently short to minimise clinical changes in participant conditions [21, 22]. VAS was also used in the second testing visit to monitor pain representing clinical condition of patients. Different VAS of < 2 cm between 1st and 2nd assessments could represent a similar clinical condition of participants [28].

The KOOS has 42 items on five subscales: pain (nine items); symptoms (seven items), activities of daily living (ADL) (17 items); sport/recreation (Sport/Rec) (five items); and knee-related QOL (four items). Each item has a five-point Likert scale response option ranging from 0 (no problems) to 4 (extreme problems), and the score for each subscale ranges from 0 (worst score) to 100 (best score). Participants were required to consider the events of the previous week when answering the questions. If at least 50% of the subscale items were answered for each subscale, a mean score was calculated. If more than 50% of the subscale items were omitted, the response was considered invalid, and no subscale score was calculated [5, 6].

The Chinese SF-36 was used to analyse the construct validity of the simplified Chinese KOOS. This questionnaire is a generic health-related outcome measure and consists of 36 items in eight domains: physical functioning; role-physical; bodily pain; vitality; general health; social functioning; role-emotional; and mental health. The questionnaire, developed by the Boston Health Research Institute, USA, provides a concise method to examine the health of the general population, aged 14 years or older [23]. The questionnaire can provide a direct quantitative indication of an individual’s health status and, because of its ease of administration, it has become the most widely used QOL assessment tool worldwide [24]. The Chinese version of SF-36 used in this study, which shows good reliability and validity, was licensed from the Institute of Social Medicine and Family Medicine, Zhejiang University School of Medicine [25].

The VAS is a one-dimensional measurement of pain intensity that has been widely used in different adult populations, including people with rheumatic diseases [26]. VAS is a continuous scale consisting of horizontal or vertical lines, usually 10 cm (100 mm). For pain intensity, the most common items on the scale are “no pain” (score of 0) and “pain as severe as possible” or “the most serious pain imaginable” (score of 100 [100 mm]). The VAS was completed by the respondents, who were asked to place a line perpendicular to the VAS line at points representing their pain intensity. The VAS takes 1 min to complete [27]. VAS management and scoring require little training and the scale is acceptable to most patients [26].

### Data acquisition and statistical analysis

Self-administered data from the simplified Chinese KOOS in the first and second rounds were used for test-retest reliability. To prevent a significant change in clinical symptoms during the study, the cut-off difference of VAS between test and retest was no more than 2 cm [28]. If the first and second VAS tests were > 2 cm, the data were not included in the test-retest reliability analysis [29]. Test-retest reliability was analysed using the intraclass correlation coefficient (ICC_3,1_) [30]. ICCs < 0.50, 0.50–0.75, 0.75–0.90, and > 0.90 were indicative of poor, moderate, good, and excellent reliability, respectively [31, 32].

Self-administered data from the first test were used to analyse internal consistency and validity. Internal consistency was used to ensure that all subscale items measuring the same construct were estimated by calculating Cronbach’s alpha (*α*). The *α* values within 0.70 to 0.95 indicated acceptable internal consistency [30]. The construct validity of the Chinese KOOS and SF-36 through convergent and discriminant validity was analysed using the Spearman rank correlation coefficient (ρ). Correlations were defined as strong (ρ ≥ 0.5), moderate (0.35 ≤ ρ < 0.5), or weak (ρ < 0.35) [30, 33].

## Results

### Translation and cross‑cultural adaptation

Forward translation mainly had forward suggestions in the functional and daily life subscale of the A10 item “rising from bed” and A15 item “going on/off the toilet”. Since A3 “rising from sitting” is in this subscale, it was suggested that A10 of the simplified Chinese KOOS should be clearly translated as “sitting up from bed”. As for item 15, Chinese people use different types of toilets, and some use squatting toilets or sitting toilets. Therefore, “going to the toilet” was suggested by the two translators. Going to the toilet can be expressed as the entire process of toilet activity regardless of the type of toilet used, and this question could be answered without ambiguity or misunderstanding.

In the pre-final version, five patients with KOA who met the inclusion criteria were interviewed face-to-face. Patients proposed matching questions and options. For example, “How stiff is your knee joint after waking up for the first time in the morning?” The answer choices of the original scale were “none”, “mild”, “moderate”, “severe,” and “extreme”. To have clearer choices of answers, it was suggested to revise the answer choices to “no stiffness”, “slight stiffness,” “moderate stiffness”, “severe stiffness”, or “extreme stiffness”. Therefore, in the simplified Chinese KOOS, adjectives were added to all the answer choices according to each question. Such modifications were approved by all the translation members and experts. After the modifications, the results of the pre-final version showed that the participants were able to complete the questionnaire successfully within 10 min without a critical question. This indicates that the simplified Chinese KOOS is easy to use and friendly for patients with KOA.

A total of 170 simplified Chinese KOOS questionnaires were distributed to trained physical therapists in 13 hospitals. Questionnaires from 158 individuals with KOA were completed and returned for analysis. Data from 128 patients were used to analyse test-retest reliability due to a ≤ 2 cm VAS difference between the first and second tests. The participants’ characteristics are shown in Table 1.

**Table 1:** Characteristics of participants with KOA

Characteristics	Completed data of the first and second tests (n = 158)	Data of ≤ 2 cm VAS difference between the first and second tests (n = 128)
Age (years)		
Mean ± SD	55.28 ± 12.41	55.57 ± 13.21
Range	28 - 86	28 - 86
**Gender (%)**		
Male	60 (38%)	52 (40.6%)
Female	98 (62%)	76 (59.4%)
**Height (cm)**	161.70 ± 8.39	162.27 ± 8.76
**Weight (kg)**	58.02 ± 9.67	59.03 ± 9.42
**BMI (kg/m** ^**2**^ **)**	22.17 ± 3.18	22.40 ± 2.99
**VAS (average ± SD)**		
First assessment	6.15 ± 2.25	5.91 ± 2.26
Second assessment	5.46 ± 2.02	5.33 ± 2.11
**Education level**		
Primary school	61 (38.6%)	50 (39.1%)
Junior middle school	61 (38.6.5)	48 (37.5%)
Technical secondary school	6 (3.8%)	8 (6.3%)
Junior college	10 (6.3%)	3 (2.3%)
Bachelor’s degree	10 (6.3%)	9 (7.0%)
Master’s degree or above	10 (6.3%)	10 (7.8%)
**Marital status**		
Unmarried	10 (6.3%)	10 (7.8%)
Married	133 (84.2%)	104 (81.3%)
Divorced	5 (3.2%)	4 (3.1%)
Widowed	10 (6.3%)	10 (7.8%)
**Residence**		
Urban area	33 (20.9%)	28 (21.9%)
County	21 (13.3%)	17 (13.3%)
Rural area	104 (65.8%)	83 (64.8%)
**Affected side**		
Left	53 (33.5%)	40 (31.3%)
Right	61 (38.6%)	47 (36.7%)
Both	44 (27.8%)	41 (32.0%)
**On set of knee OA (years)**		
< 1 year	46 (29.1%)	37 (28.9%)
1–3 years	37 (23.4%)	33 (25.8%)
3–5 years	24 (15.2%)	21 (16.4%)
5–7 years	10 (6.3%)	7 (5.5%)
≥ 7 years	41 (25.9%)	30 (23.4%)

KOA, knee osteoarthritis; SD, standard deviation; VAS, visual analogue scale; OA, osteoarthritis

### Test-retest reliability

The test-retest reliability between the first and second measurements was 0.808–0.976, which indicates that the simplified Chinese KOOS has good to excellent test-retest reliability (Table 2).

**Table 2:** Test-retest reliability of the simplified Chinese KOOS in five subscales (n = 128 patients, separated with an interval 5–7 days)

KOOS score	Pain	Symptoms	ADL	Sport/Rec	QOL
First assessment (mean ± SD)	59.97 ± 18.33	61.45 ± 20.26	66.52 ± 21.31	42.30 ± 27.26	47.45 ± 18.81
s assessment (mean ± SD)	63.45 ± 18.15	62.95 ± 18.44	68.00 ± 19.61	45.70 ± 24.61	52.15 ± 16.61
ICC (3,1)	0.926	0.914	0.976	0.946	0.808
95% CI	0.906–0.944	0.890–0.934	0.970–0.982	0.931–0.959	0.753–0.854
P	< 0.001	< 0.001	< 0.001	< 0.001	< 0.001

ICC, intraclass correlation coefficient; CI, confidence interval; SD: Standard deviation; ADL: Activities of daily living; Sport/Rec: Sports and recreation function; QOL, knee-related quality of life. ICCs < 0.50, 0.50–0.75, 0.75–0.90, and > 0.90 were indicative of poor, moderate, good, and excellent reliability, respectively

### Internal consistency

The overall coefficient of the simplified Chinese KOOS was 0.973, which suggests that the internal consistency reliability was satisfactory. The internal consistency of the five subscales is shown in Table 3.

**Table 3:** Internal consistency of the KOOS subscales

KOOS Subscales (number of items)	Cronbach’s *α* coefficients (n = 158)
Pain (9)	0.902
Symptoms (7)	0.822
ADL (17)	0.970
Sport/Rec (5)	0.944
QOL (4)	0.757
Total (42)	0.973

ADL, Activities of daily living; Sport/Rec, Sports and recreation function; QOL, knee-related quality of life. The *α* values within 0.70 to 0.95 indicated acceptable internal consistency

### Construct validity

In the current study, the SF-36 was preferred as the comparative scale for structural validity. Table 4 presents the results.

**Table 4:** Spearman’s ρ correlation between the simplified Chinese KOOS and Chinese SF-36 scores

		KOOS				
	**Subscale**	Pain	Symptoms	ADL	Sport/Rec	QOL
**SF-36**	Physical function	**0.537** ^**^	**0.380** ^**^	**0.628** ^**^	**0.619** ^**^	**0.484** ^**^
	Role-physical	**0.272** ^**^	*0.136*	**0.296** ^**^	**0.335** ^**^	**0.369** ^**^
	Bodily pain	**0.435** ^**^	**0.275** ^**^	**0.437** ^**^	**0.436** ^**^	**0.483** ^**^
	General health	**0.276** ^**^	0.165^*^	**0.304** ^**^	**0.329** ^**^	**0.342** ^**^
	Vitality	**0.262** ^**^	*0.151*	**0.276** ^**^	**0.298** ^**^	**0.299** ^**^
	Social function	**0.274** ^**^	0.201^*^	**0.272** ^**^	0.213^*^	0.181^*^
	Role-emotional	**0.312** ^**^	0.180^*^	**0.333** ^**^	**0.274** ^**^	**0.341** ^**^
	Mental health	**0.336** ^**^	**0.225** ^**^	**0.355** ^**^	**0.302** ^**^	**0.310** ^**^

Strong correlations in bold type, moderate correlations in regular type, weak correlations italics; strong (ρ ≥ 0.5), moderate (0.35 ≤ ρ < 0.5), or weak (ρ < 0.35) [30, 34]

*Correlation is significant at the 0.05 level (2-tailed)

**Correlation is significant at the 0.01 level (2-tailed)

SF-36, Short Form-36 Health Survey; ADL, Activities of daily living; Sport/Rec, Sports and recreation function; QOL, knee-related quality of life

## Discussion

We aimed to cross-culturally translate the original English KOOS to the simplified Chinese KOOS and to investigate the psychometric properties of individuals with KOA in mainland China. Forward translation mainly had suggestions in the functional and daily life subscale of the A10 item “rising from bed” and A15 item “going on/off the toilet”. To obtain clearer answers, adjectives were added to all the answer choices according to each question. Questionnaires from 158 individuals with KOA were completed and returned, demonstrating an effective recovery rate of 93%.

Currently, the Hong Kong and Singaporean Chinese versions are available on the official KOOS website. The Chinese version from Singapore has the same translation and cross-cultural adaptation as that of the English version. However, the Hong Kong Chinese version was derived from the Chinese version from Singapore. As Chinese and Cantonese languages are different, the items A16, A17, P2, SP4, and Q4 were discussed because of the expressions of “intensity of housework” and “knee joint” [16]. These words have been replaced by culturally related Cantonese translations, which are preferred in the Hong Kong version. In our study, we used an original English version of the KOOS for translation and cross-cultural adaptation to the simplified Chinese KOOS for Chinese people in mainland China.

Previous studies have demonstrated that the KOOS can be applied to young people and patients with various knee joint problems [5, 6]. In the current study, the average age of the participants was 55.28 years, ranging from 28 to 86 years. This shows that the simplified Chinese KOOS is understandable to Chinese people of a wide range of ages. In 2017, a study of pooled data from 26 unique cohorts [27] revealed that all subscales demonstrated an adequate test-retest reliability ICC range of 0.85–0.9. In the OA group, the ICC values for each subscale were pain (0.85–0.92), symptoms (0.83–0.91), ADL (0.84–0.93), Sport/Rec (0.73–0.89), and QOL (0.78–0.88). The ICC values of the current study were 0.926, 0.914, 0.976, 0.946, and 0.808, respectively (Table 4). A cut-off ICC of 0.70 was proposed as an acceptable reliability [30]. This indicates that the simplified Chinese KOOS has very good reliability for measurement over time across other versions.

An examination of internal consistency is required for validation studies to ensure that all subscales measure the same construct. In 2016, Collins et al. [21] reported that the internal consistency of the total value of Cronbach’s *α* of 25 KOOS versions was between 0.70 and 0.95. In the OA group, the Cronbach’s *α* values of each subscale were pain (0.85–0.92), symptoms (0.83–0.91), ADL (0.84–0.93), sport/recreation (0.73–0.89), and QOL (0.78–0.88). The Cronbach’s *α* values in the current study were 0.902, 0.822, 0.970, 0.944, 0.757, and 0.973, respectively. The total Cronbach’s *α* was 0.973. This indicates that the simplified Chinese KOOS is good for all items of the subscale measurement, similar to the other language versions.

Normally, construct validity is required to examine the validity of a questionnaire assessment. The current study examined construct validity by comparing the simplified Chinese KOOS and the SF-36. A high correlation (ρ ≥ 0.5) of convergent validity was expected between KOOS pain subscale and SF-36 bodily pain subscale, and KOOS ADL/Sport/Rec/QOL subscale and SF-36 physical functioning subscale. Spearman’s ρ correlations of the current study were as expected, except between KOOS pain subscale and SF-36 bodily pain subscale, and between KOOS QOL subscale and SF-36 physical functioning subscale, which showed moderate correlations. For discriminant validity (ρ < 0.5), the findings showed an expectation of low correlation between KOOS-all subscales except with ADL and SF 36-Mental health (Table 4). The SF-36 and EQ-5D were used to test the construct validity of the Singaporean Chinese version of the KOOS. The results showed that KOOS-symptom had the best construct validity and KOOS-Sport/Rec had the worst construct validity. The current study supports previous studies of the Hong Kong KOOS version [16] and the study by Zhang et al. [35].

Previous research studied the KOA group and reported the convergence and discriminant validity of the Hong Kong KOOS [16] by comparing it with the Chinese version of the Concise Short Form 12 (SF-12) health survey, the China Modified Barthel Index (C-MBI), and VAS Pain. The results of the Hong Kong version showed a linear relationship between the KOOS-symptom and SF-12 physical component summary subscales; the KOOS-Sport/Rec score weakly correlated with the SF-12 mental component summary scale. The results of our study were similar with Hong Kong version that the KOOS symptom subscale was moderately correlated with the SF-36 physical function subscale (ρ = 0.380, p < 0.001) and the KOOS-Sport/Rec subscale was weakly correlated with the SF-36 mental health subscale (ρ = 0.302, p < 0.001). To determine discriminant validity, we hypothesized that weak correlation would be observed between KOOS symptom subscale and SF-36 bodily pain subscale, and KOOS all subscales and SF-36 mental health subscale. In Table 4, our hypotheses were supported except with KOOS ADL subscale and SF-36 mental health subscale.

In another study of Hong Kong KOA patients by Cheung et al. [36], WOMAC and SF-36 were used to study the construct validity of the KOOS, and a comparison of our study with the results of this study revealed that the Sport/Rec and pain subscales of KOOS were highly correlated with the physical function subscale of the SF-36, the KOOS-QOL subscale and the SF-36 role-physical subscale showed a moderate correlation, and the remaining four subscales of KOOS showed a weak correlation with the SF-36 role-physical subscale. In the study by Zhang et al. [35], the KOOS was studied in anterior cruciate ligament reconstruction patients using a Chinese version converted from the Singaporean version and validated for construct validity using the SF-36. These studies found moderate to strong correlations between all subscales of KOOS and the SF-36 physical function subscale and weak correlations between the SF-36 general health, social function subscales, and all subscales of KOOS.

The current study had some limitations. First, participants with KOA in Yunnan Province were recruited for the psychometric testing. To confirm the benefits of other knee injury conditions, further studies should include various knee conditions. Secondly, the A10 item “sitting up from bed” of the simplified Chinese KOOS of the current study was not the same as the original KOOS. The original developer asked that the A10 item should be understood as “from lying down to standing up”. However, the authors believed that this did not affect the psychometric properties of the simplified Chinese KOOS at the overall and subscale levels. In the published final version of the simplified Chinese KOOS, the A10 item was corrected as “from lying down to standing up”.

## Conclusions

The simplified Chinese KOOS in the current study comprises five subscales (42 items), similar to the original English version. After scientific and systematic simplified Chinese translation procedures, the results were easily understanding and reliable, and the reliability and validity of the KOOS version were good. All indicators met the methodological standards and acceptable psychometric properties. Therefore, in clinical and research fields, the simplified Chinese KOOS in the current study can be used as a self-administered measurement for individuals with KOA in China.

## Data Availability

The data that support the findings of this study are available from the corresponding author upon reasonable request.

## Abbreviations

KOOS: Knee Injury and Osteoarthritis Outcomes Score
KOA: Knee osteoarthritis
ICC: Intraclass correlation coefficient
QOL: Quality of life
WOMAC: Western Ontario and McMaster Universities Osteoarthritis Index
VAS: Visual analogue scale
ACR: American College of Rheumatology
ADL: Activities of daily living
CI: Confidence interval
SD: Standard deviation
Sport/Rec: Sports and recreation function
SF-12: Short Form 12
C-MBI: China Modified Barthel Index
